# scikit-matter : A Suite of Generalisable Machine Learning Methods Born out of Chemistry and Materials Science

**DOI:** 10.12688/openreseurope.15789.1

**Published:** 2023-05-16

**Authors:** Alexander Goscinski, Victor Paul Principe, Guillaume Fraux, Sergei Kliavinek, Benjamin Aaron Helfrecht, Philip Loche, Michele Ceriotti, Rose Kathleen Cersonsky

**Affiliations:** 1 Laboratory of Computational Science and Modeling (COSMO), Institute of Materials, Ecole Polytechnique Federale de Lausanne, Lausanne, Vaud, 1015, Switzerland; 2 Pacific Northwest National Laboratory, Richland, WA, 99352, USA; 3 Department of Chemical and Biological Engineering, University of Wisconsin-Madison, Madison, Wisconsin, 53706, USA

**Keywords:** Python, feature selection, sample selection, PCovR, KPCovR, feature reconstruction, directional convex hull

## Abstract

Easy-to-use libraries such as scikit-learn have accelerated the adoption and application of machine learning (ML) workflows and data-driven methods. While many of the algorithms implemented in these libraries originated in specific scientific fields, they have gained in popularity in part because of their generalisability across multiple domains. Over the past two decades, researchers in the chemical and materials science community have put forward general-purpose machine learning methods. The deployment of these methods into workflows of other domains, however, is often burdensome due to the entanglement with domain-specific functionalities. We present the python library scikit-matter that targets domain-agnostic implementations of methods developed in the computational chemical and materials science community, following the scikit-learn API and coding guidelines to promote usability and interoperability with existing workflows.

## Plain-language summary

Numerous data-driven and machine-learning studies rely on the library scikit-learn, a package providing implementations and application interfaces to a collection of generally applicable machine learning (ML) methods. With scikit-matter (skmatter) we extend current methods, focusing on those that are actively used in the field of computational science and modeling of materials and chemical systems. We aim to provide users with the ability to seamlessly include these methods in their ML workflows by implementing them in compliance with the scikit-learn API and coding guidelines.

## Introduction

While machine learning (ML) algorithms are applied in a wide variety of fields, the relative importance of different aspects of workflows can vary widely between disciplines. For those who apply ML to predict physical or chemical properties, there is an increased emphasis on engineering and understanding numerical features from the underlying physical entities—namely, atoms and their relative position in the structure or molecule—in a format compatible with ML pipelines ^1– 4^. Engineering these features is a rich sub-discipline of machine learning unto itself, and thus several best practices have been established for analysing the resulting representations, including a reduction of redundant information in features and samples ^5– 7^, comparing different featurisations on datasets ^8, 9^, and analysing their reduced manifolds ^10^. These methods are, however, often inextricably linked to the libraries computing these representations ^11– 13^ and are not available to a wider audience outside of these sub-communities. The objective of the open-source library scikit-matter is to make these ML methods accessible to a wider community by following the scikit-learn API and coding guidelines, and by treating the features as agnostic to their domain. It not only serves as a conduit between featurisation software for comparing alternative descriptors, but allows a ‘plug-and-play’ of these methods into any workflows, irrespective of the representation or field of use.

In this text, we will review several major modules in scikit-matter: 1) Feature Reconstruction Measures (Page 3), used to assess the mutual information contained in two separate representations of the same dataset, 2) Linear and Kernelized Principal Covariates Regression (Page 5), used to construct a new set of features designed to correlate with one or more target properties, 3) Farthest Point Sampling, CUR, and their PCovR-Adaptations (Page 11), methods used to sub-select features and/or samples for machine learning problems, and 4) the Directional Convex Hull Construction Page), used to identify data points that sit on a bounding manifold of a dataset in a particular direction.

We demonstrate the use of these methods in contexts both inside and outside the chemical domain. We first revisit a dataset originally published in Engel *et al.* ^14^ and available publicly via the Materials Cloud Archive ^15, 16^. This dataset contains theoretical and known ice structures, *i.e.* 15’869 “reasonable” crystal structures with hydrogen and oxygen in a 2:1 ratio, their lattice energies (in eV/molecule) and densities (in g/cm ^3^). We have augmented this dataset to include the atomic Mulliken charges, as computed with DFTB+ ^17^, in units of elementary charges [e].

We also show the utility of these methods beyond the chemical sciences by employing the World Health Organization statistics on life expectancy ^18^, curated from the open repository from the World Bank. This dataset contains 2,020 data points, each representing a different country during a given year, reporting variables pertaining to the population size ^19^, gross domestic product (GDP) ^20^, health-based ^21^ and education-based ^22^ spending, prevalence of HIV / AIDS ^23^ and tuberculosis ^24^, immunisations ^25, 26^, and undernourishment of the country’s population ^27^.

## A Quick Guide to the Smooth Overlap of Atomic Positions (SOAP) Representation

For the ice dataset we use the Smooth Overlap of Atomic Positions (SOAP) representation to numerically encode our chemical data ^28^. As the SOAP formalism is not well-known outside the atomistic machine learning field, here we give a quick and simplified overview of the concepts and terminology associated with this data representation.

Numerous electronic and chemical properties are determined by the spatial relationship between a “central” atom and its nearest neighbouring atoms ^29^. For most properties, the importance of neighbouring atoms decays with their distance from our central atom. It is therefore worthwhile to only represent the neighbourhood around each atom up to a cutoff. In the SOAP representation, each atom in the neighbourhood is represented by a Gaussian density. A finite set of numerical descriptors of all densities in the neighbourhood is then used as features.

However, in order to make efficient use of our data, we need to incorporate the symmetries of our system, as many physical properties do not change under rotations and translations. We therefore use the neighbourhood centered on each atom *i*, known as *atomic density ρ _i_*, to remove the dependency on the origin of our system ^6^. The neighbourhood is described by a combination of radial and spherical functions to form a basis for the expansion. One can choose one of many functional forms for radial bases ^4, 28, 30^, and any number of basis functions to do this expansion. The number of basis functions determines the number of features. In general, a higher amount of features corresponds to a higher “resolution” of the neighbourhood.

The set of expansion coefficients of *ρ _i_* in this basis results in something known as *density coefficients* for each central atom *i*. We can take the correlation of these coefficients to build descriptors corresponding to two-body correlations $\rho_{i}^{⊗1}$ , three-body correlations $\rho_{i}^{⊗2}$ , or higher. Here, the superscript corresponds to the *ν* ^th^-neighbour correlation order expansion, where a two-body correlation is the first-order expansion. We often write features symmetrised over rotations as $\bar{\rho_{i}^{⊗v}}$ specifically calling $\bar{\rho_{i}^{⊗1}}$ the “Radial Spectrum” and $\bar{\rho_{i}^{⊗2}}$ the “Power Spectrum”.

A full explanation of the SOAP formalism is contained in Bartók *et al.* ^28^ and Willatt *et al.* ^6^. SOAP is not the only popular machine-learning representation in chemical sciences, and a full review of many of the different physics-informed representations is available at Musil *et al.* ^4^.

In line with its goal of being disentangled from domain-specific components, scikit-matter itself does not compute these atomic descriptors, and instead takes as input matrices corresponding to descriptors computed by prominent software such as librascal ^4^, QUIP ^31– 33^, and DScribe ^34^. For the purposes of testing and examples, scikit-matter does contain minimal datasets, each including a small set of molecules or materials, a suitable featurisation, and a set of properties ^35, 36^.

## Methods contained in scikit-matter

### Feature reconstruction measures

In order to compare two separate sets of features, one can employ regression errors to quantify the mutual relationships of different forms of featurisations, as demonstrated in Goscinski *et al.* ^36^. We determine this error, or the feature reconstruction measure (FRM), by reconstructing one set of features from the other with a constrained transformation, where different constraints express different types of relationships between the two sets of features.

Say we have a dataset that is represented in full detail by a representation Θ, and we want to assess the amount of information lost by using an alternate representation *ℱ*. We can check the detail contained in *ℱ* by computing FRM( *ℱ*, Θ), where FRM = 0 corresponds to a perfect reconstruction with no loss, and FRM ≈ 1 denotes a complete information loss wrt. Θ. However, it is rare that there exists a ground-truth representation Θ and we are more commonly comparing two likely-imperfect representations *ℱ* and . In this case, we compute FRM( *ℱ*, ) and FRM( , *ℱ* ). The feature set that results in the higher reconstructive measure is considered higher in information capacity ( *e.g.* if FRM( *ℱ*, ) > FRM( , *ℱ* ), then *ℱ* is the more information-rich feature set).

**Figure 1.**
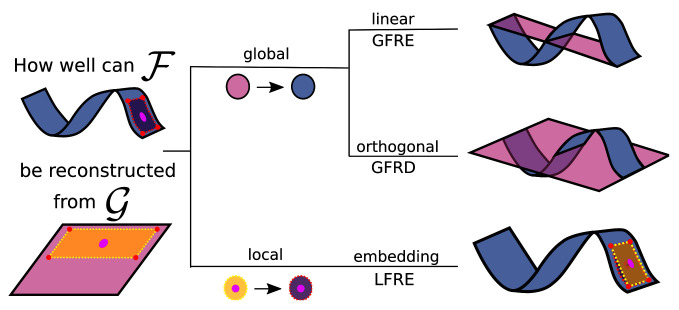
The different forms of feature reconstructions to assess two feature spaces (blue and pink) describing the same dataset. Here, we are reconstructing the curved manifold (blue) using the planar manifold (pink), as it is often the case to approximate a complex manifold with a simpler alternative. The area framed by the dotted line is an example for a local neighbourhood of one sample (the pink dot) that enables the reconstruction of nonlinearities. (Top) The linear transformation used in the global feature reconstruction error (GFRE). (Middle) The orthogonal transformation used in the global feature reconstruction distortion (GFRD). (Bottom) A local linear transformation of a neighbourhood used in local feature reconstruction error (LFRE). On the right, the reconstructions of the manifold are drawn in pink together with the curved manifold in blue. The measures correspond to the root-mean-square difference between the reconstructed and curved manifold.

In Figure 1 we show a schematic of the different FRMs contained in scikit-matter. The simplest FRM is the global feature reconstruction error (GFRE), expressed as the linearly decodable information, as given by performing ridge regression between the two sets of features. The global feature reconstruction distortion (GFRD) constrains the transformation to be orthogonal to demonstrate the deformation incurred by transforming between the two feature spaces.

Extending the analysis to non-linear dependencies, the local feature reconstruction error (LFRE) applies the ridge regression for each point locally on the *k*-nearest neighbours. These unsupervised methods are of particular use in assessing the hyperparameters of ML descriptors ^9^ and have been employed to compare the efficiency of different basis sets in encoding geometrical information ^4, 37^.

### Implementation

The FRMs differ in two aspects: the locality of the reconstruction (global or local) and the constraints of the regression. In each FRM, the two feature sets are partitioned into training and testing sets. We standardise the features of *ℱ* and individually, then we regress the features of *ℱ* onto to compute the errors. In the global measures, we use the entire dataset for the reconstruction, whereas in the local measure we perform a regression for each sample on the set of the *k*-nearest points within *ℱ*. The number *k* is given by the user with the parameter n_local_points. The reconstruction *error* is by default computed using a 2-fold cross-validation and ridge regression as estimator. As most interesting applications use large feature vectors, we implemented a custom RidgeRegression2FoldCV in the skmatter.linear_model module to improve computational efficiency. For the reconstruction *distortion* we use orthogonal regression as implemented in OrthogonalRegression in the skmatter.linear_model module.

**Workflow ** The FRMs can all be called similarly:


from skmatter.metrics import
    global_reconstruction_error as GFRE
gfre = GFRE(F, G)

from skmatter.metrics import
    local_reconstruction_error as LFRE
lfre = LFRE(F, G, n_local_points=10)


It is also possible to specify general feature scalers and estimators in any of the FRM classes, in which case the invocation looks like:


from skmatter.metrics import
    global_reconstruction_error as GFRE
    
gfre = GFRE(F, G, scaler=my_scaler,
             estimator=my_estimator)


### Use Case: Determining the number of radial basis functions necessary for representing neighbourhood shells

**Figure 2.**
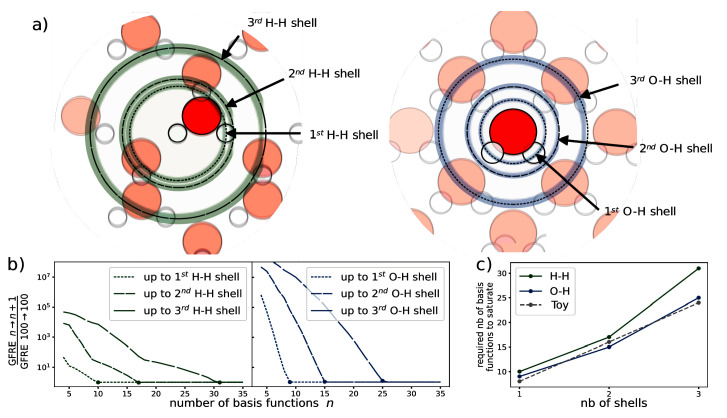
The relationship between number of radial basis functions and the resolution of each neighbourhood shell in water. **a**) The different atomic shells present in the ice dataset. **b**) The convergence of the GFRE for an incremental change in the number of basis functions normalised by the numerical accuracy of the linear system solver. The numerical accuracy of the solver is determined by computing the GFRE for the identity relation for 100 basis functions. **c**) The number of basis functions required to resolve up to the n ^th^ neighbour shell.

In common frameworks for machine learning potentials, each atomic neighbourhood is represented by expanding the many-body correlation information over a set of basis functions ^4^. The resolution of this expansion varies greatly based upon the type of dataset and choice of basis. In this use case, we analyse the number of radial basis functions required to resolve each neighbour shell from a central atom. Here, “shell” refers to a spherical shell that corresponds to a peak in the radial distribution function as sketched out in Figure 2a). For a given choice of basis functions, we say that the representation is *converged* when adding new bases in the series yields no new information. In other words, we can say that the number of basis functions *n* has converged when the GFRE *n* → *n* + 1 saturates. Here we show the convergence behaviour of a PCA-optimised ^1^ basis set ^37^ expanded on the “Radial Spectrum”, showing the number of basis functions needed to resolve each neighbour shell up to numerical accuracy of the linear system solver within the ice dataset. The numerical accuracy of the solver is computed with GFRE for the identity relationship for 100 basis function.

For this dataset, we determine two neighbour shells for the species pairs H-H and O-H (demonstrated in Figure 2(a)). With each additional shell, we need a greater number of basis functions, as shown in Figure 2(c). We see a near-linear relationship between the number of shells considered and the number of basis functions needed to obtain convergence. In fact, using a toy dataset with equidistant shells and a uniform distribution of atoms in each shell, we recover a perfect linear relationship. Thus, the number of basis functions needed to obtain similar resolutions scales linearly with the number of shells. The difference between these results and those of the toy dataset can be explained by the irregularly-spaced shells and the non-uniform number of atoms across shells in the ice dataset.

### Linear and non-linear principal covariates regression

Often, one wants to construct new ML features from their current representation in order to compress data or visualise trends in the dataset. In the archetypal method for this dimensionality reduction, principal components analysis (PCA), features are transformed into the latent space which best preserves the variance of the original data. *Principal Covariates Regression* (PCovR), as introduced by de Jong and Kiers ^38^, is a modification to PCA that incorporates target information, such that the resulting embedding could be tuned using a mixing parameter *α* to improve performance in regression tasks ( *α* = 0 corresponding to linear regression and *α* = 1 corresponding to PCA). Helfrecht *et al.* ^10^ introduced the non-linear version, *Kernel Principal Covariates Regression* (KPCovR), where the mixing parameter *α* now interpolates between kernel ridge regression ( *α* = 0) and kernel principal components analysis (KPCA, *α* = 1) ^39^.

**Figure 3.**
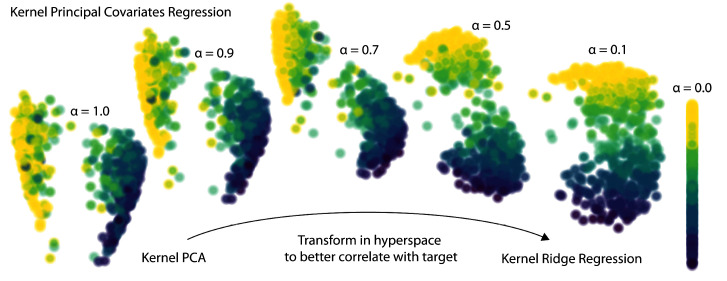
The evolution of latent-space projections and regressions as the mixing parameter *α* goes from 1 (Kernel PCA) to 0 (Kernel Ridge Regression) in Kernel PCovR. This procedure transforms the latent space projection to minimise combined KPCA and KRR loss. Typically, a value of *α* = 0.5 yields a balanced projection and can be used to construct insightful feature-property maps.

The *α* parameter determines how much emphasis to give either the regression performance or feature reconstruction. As shown for a toy dataset in Figure 3, a KPCovR of *α* = 1.0 will replicate the results of a Kernel PCA (weighted entirely on the reconstruction task), whereas *α* = 0.0 contains the regression weights as features. Typically, a value of *α ≈* 0.5 yields the most qualitatively insightful results, provided that the features and targets are properly normalised.

### Implementation

In a PCA decomposition, we obtain a latent-space projection by taking the singular value decomposition of the feature matrix **X**,


$$X=U_{K}\sum U_{C}^{T}\;(1)$$

where **U**_*K*_ and **U**_*C*_ are the eigenvectors of the Gram matrix ( **XX** ^*T*^) and covariance matrix **X** ^*T*^ **X** and Σ are the singular values. In PCovR, we instead use the eigendecomposition of a modified Gram or covariance matrix, for example mixing **XX** ^*T*^ with the outer product of our predicted targets **ŶŶ** ^*T*^. As covered by de Jong and Kiers ^38^, it is useful to build this decomposition on the approximated target values. In scikit-matter, this is done by providing the predicted targets **Ŷ** or a scikit-learn-style estimator with which to regress **X** onto **Y**.

Similarly, KPCovR constructs a modified kernel matrix, replacing the Gram matrix **XX** ^*T*^ with a user-selected kernel (with kernel-building capabilities and an API consistent with scikit-learn) and requires a kernel regressor to obtain the approximated **Ŷ**.

**Workflow ** For example, a typical invocation of KPCovR is:


import skmatter.kernel_ridge.KernelRidge
import skmatter.decomposition.KernelPCovR

kernel_params = dict(kernel="rbf",
                        gamma=1)

regressor = KernelRidge(**kernel_params)

kpcovr = KernelPCovR(
                        mixing=0.5,
                        n_components=2,
                        regressor=regressor,
                        **kernel_params
                       )
kpcovr .fit(X, Y)

T = kpcovr.transform(X)
Yp = kpcovr.predict(X)


where **X** and **Y** are standardised matrices containing our features and target properties, respectively. For linear PCovR, regressor can be set to be any linear regression object within scikit-learn.

### Use Case: Mapping the charge of oxygen in ice

We build a linear PCovR map using as **X** the 3-body SOAP representations reported in Engel *et al.* ^14^ and as targets **Y**, the Mulliken charges ^2^ (in units of *e*) for each of the water atoms in a subset of the ice structures.

**Figure 4.**
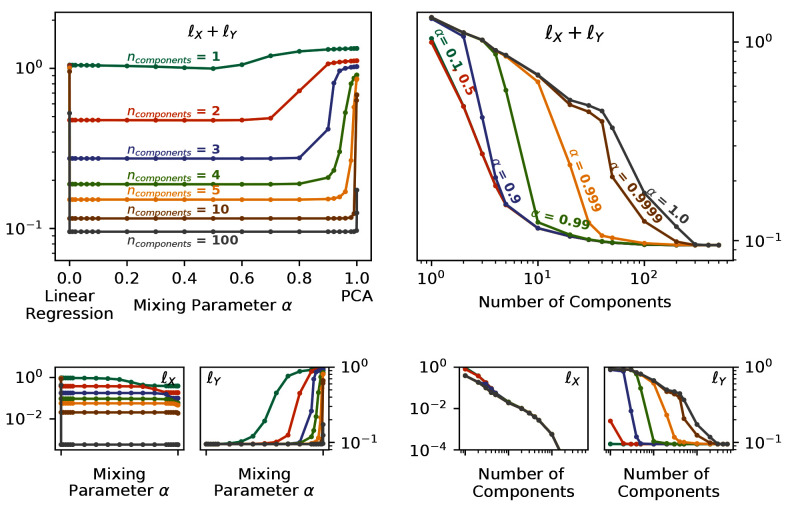
Reconstruction and Regression Errors for the PCovR Mappings. In each case, we report the unitless *l* ^2^ loss for multiple values of α and number of components for reconstruction $\ell_{X}\equiv {\left\|X-\hat{X}\right\|}^{2}$ and regression of the Mulliken charges $\ell_{Y}\equiv {\left\|Y-\hat{Y}\right\|}^{2}$ , both the sum (top) and separately (bottom). (left) Plotted against the Mixing Parameter *α*. (right) Plotted against the Number of Components.

By changing the mixing parameter *α*, we directly specify the attention that PCovR (or KPCovR) gives to the learning tasks of the target properties. In Figure 4, we show the performance of a PCovR mapping at both reconstructing the original SOAP vectors ( $\ell_{Y}\equiv {\left\|X-\hat{X}\right\|}^{2}$ ) and regressing the charges ( $\ell_{Y}\equiv {\left\|Y-\hat{Y}\right\|}^{2}$ ). Here we use a fixed regularisation constant for comparability between different numbers of components and mixing parameters.

While all latent-space projections approach the same loss with increasing dimensionality, when using fewer components, the effect on the regression loss _*Y*_ is much larger than on the reconstruction loss _*X*_, as shown by the near-flat curves in _*X*_ across the mixing parameter. Conversely, the change in _*Y*_ across *α* can be quite large (as also demonstrated by the poor regression saturation to *α* = 0.999 in the top-right panel). You will also notice, particularly in the top-left panel, that the errors increase asymptotically in the extreme cases ( *α* = 0, *α* = 1). Thus in the majority of cases, an intermediate value will not only provide a better combined error, but also better performance in regression tasks while imparting minimal impact on the reconstruction task.

**Figure 5.**
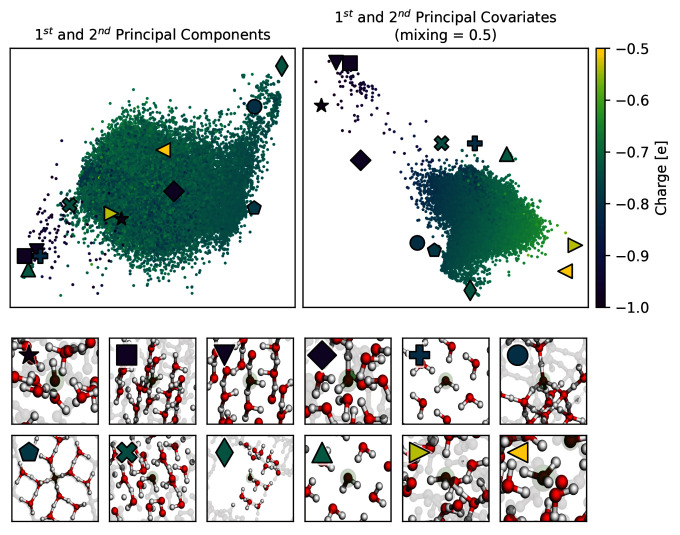
A Comparison of PCovR and PCA maps of Oxygens in Ice Structures. Both the upper maps show the first two components (left) or covariates (right) of the oxygen environments, coloured by the Mulliken charge. We have highlighted several extreme examples with special markers, corresponding to the markers also shown in the upper left corner of each inset.

We also often choose a mixing parameter of *α* = 0.5 for visualisation tasks to weight equally the reconstruction and regression tasks. The resulting map, shown in the right side of ( Figure 5), delineates our oxygen environments based upon chemical similarity and our target charges, and from here we can clearly see the separation of oxygens in hydroxide (⋆, □, ᐁ, ⋄), hydronium (⊲, ⊳), and different arrangements of water, as shown in the insets below the maps. This is not true in the corresponding PCA (left), which is unable to distinguish these very different environments.

### Use Case: Reducing the dimensions of the WHO dataset to predict life expectancy

**Figure 6.**
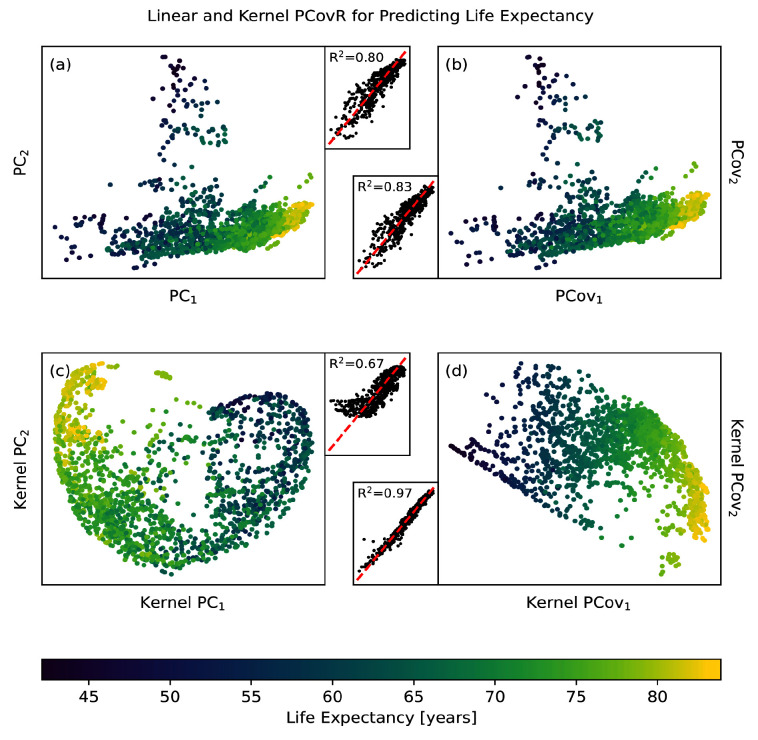
Linear and Non-linear Principal Components Analysis and Principal Covariates Regression Applied to the WHO Dataset. Here we show the resultant map and parity plot for ( **a**) PCA, ( **b**) PCovR at *α* = 0.5, ( **c**) KPCA, and ( **d**) KPCovR at *α* = 0.5 based on 13 statistical variables in order to predict life expectancy for a given country. In each case, we used the same standardised variable matrix to determine a two-dimensional latent space mapping (shown in the map) that was then fed into an appropriate regularised regression model, yielding the predictions in the parity plot. Regressions were all computed using the same 90/10 train/test split, with regression results reported for only the testing split.

The variables in the WHO dataset suffice to predict the life expectancy with an R ^2^ score of 0.87 on an off-set testing set with cross-validated ridge regression. When we use traditional PCA to reduce this set of variables to two components, our accuracy in prediction drops to R ^2^ = 0.81 ( Figure 6(a)). This is marginally raised by turning to PCovR, where regressing on the first two covariates gives R ^2^ = 0.83 ( Figure 6(b)).

The most dramatic results come from using non-linear methods. Employing kernel ridge regression with an optimised RBF kernel on the original features raises our maximum accuracy to R ^2^ = 0.96. Using the same kernel parameters to choose two features via kernel PCA reduces our accuracy to R ^2^ = 0.57 ( Figure 6(c)); however, by doing the same with kernel PCovR we sacrifice little in accuracy (R ^2^ = 0.96, Figure 6(d)). In other words, we are able to obtain nearly the same regression performance using two covariates determined by KPCovR as we did with the full kernel. This example, from start-to-finish, is available on the scikit-matter documentation via https://scikit-matter. readthedocs.io/en/v0.1.4/read-only-examples/FeatureSelection-WHODataset.html.

These kernel principal covariates can then be used to perform correlative analysis with the original demographic or statistical features, as was demonstrated by Cersonsky *et al.* ^40^ for medical diagnostics in the context of stillbirth outcomes.

### Feature and sample selection

In this section, we will detail two very different classes of selectors: 1) Farthest Point Sampling, CUR, and their PCovR adaptions, which are flexible score-based greedy selectors ^3^ that can be employed for either feature or sample selection; and 2) the directional convex hull, a non-greedy sample selection algorithm which is primarily used in chemical thermodynamic analyses.

The feature and sample selection methods contained in scikit-matter are both programmatically alike and contextually different. Therefore, we established a consistent API to allow users to apply these similar concepts in their different contexts, and we will start by reviewing this common implementation.

### Implementation

The classes are exposed to the user via skmatter.feature_selection and skmatter.sample_selection. Each selector is passed an input of **X** (and **Y**, when appropriate) to a fit function. This fit function will perform the sub-selection up until the desired number of selections (initialisation parameter n_to_select), the algorithm has terminated, or some score threshold has been reached (initialisation parameter score_threshold). Once the fitting is complete, the selector will contain the indices of the selections (relative to input matrices) in a member variable selected_idx_. The scores that evaluate all points in the context of these selections are available via the score function.

### Farthest point sampling, CUR, and their PCovR-adaptations

Farthest point sampling (FPS) and CUR decomposition are available in unsupervised ^41, 42^ or hybrid (PCovFPS and PCovCUR) ^7^ variants for both feature and sample selection. In the hybrid variant, similar to the dimensionality reduction techniques discussed in Section "Linear and non-linear principal covariates regression" on Page 5, a parameter *α* is used to weight the supervised and unsupervised contributions in the covariance (for feature selection) or the Gram matrix (for sample selection) in the range from 0 to 1, with *α* = 1.0 directly corresponding to the unsupervised version of each algorithm published in Imbalzano *et al.* ^42^

**FPS** In Farthest Point Sampling (FPS), we start with a random point, and at each iteration select the point that maximises the distance to the previously selected set. This min-max distance is traditionally called the Hausdorff distance, which serves as the FPS scoring metric. The unsupervised variant uses the Euclidean distance as the default measure for the Hausdorff distance. The hybrid variant incorporates regression weights from an estimator into the distance calculation based upon the relationship between Euclidean distance and the corresponding covariance or Gram matrices of the input matrix.

Computing the Hausdorff distance, in any variable space, constructs an implicit Voronoi tessellation ^43^ that can be exploited to reduce the amount of distance calculations throughout the selection procedure. At each iteration, only the selected points at the boundaries of the Voronoi polyhedra need to be considered for new distance calculations. We have implemented a FPS variant computation of Voronoi skmatter.sample_selection.VoronoiFPS which results in large speedups for sample selections in dense datasets, where the effects of this algorithm can be largest. Benchmark results comparing classical FPS and the Voronoi version can be found in the supporting information of Cersonsky *et al.* ^7^

**Workflow ** A typical workflow using FPS looks like this:


import skmatter.feature_selection.FPS
selector = FPS(
                 n_to_select=4,
                 progress_bar=True,
                 score_threshold=1E-12,
                 initialize=[0,1,2]
                )
selector.fit(X)


where the parameter n_to_select refers to the number of selections to be made, progress_bar signifies whether to show a tqdm-style progress bar ^44^, score_threshold is the score to terminate the selection at (potentially before n_to_select), and initialise signifies the index or indices with which to start the selection procedure.

**CUR** selects features or samples based upon an “importance score” *π*, defined as the magnitude of a feature or sample’s projection along the first *k* principal components (unsupervised) or covariates (hybrid PCovCUR) of the overall dataset ^7, 41, 42^. *π* serves as the scoring metric for CUR. In the iterative implementation, at each iteration, the remaining features/samples are orthogonalised to the previous selection, as this ultimately minimises the mutual information in each subsequent selection ^42^.

**Workflow ** A typical workflow using CUR looks like this:


import skmatter.feature_selection.CUR
selector = CUR(
                 n_to_select=4,
                 progress_bar=True,
                 score_threshold=1E-12,
                 k=1,
                 recompute_every=1
                )
selector.fit(X)


where, similar to FPS, the parameter n_to_select refers to the number of selections to be made, progress_bar signifies whether to show a tqdm-style progress bar, and score_threshold is the score to terminate the selection at (potentially before n_to_select). k is the number of components or covariates to consider, and the parameter recompute_every indicates after how many iterations to orthogonalise the remaining features or samples. The latter can speed computation (at the cost of including multiple redundant features or samples), as orthogonalising and recomputing the *π* score are the most costly steps in the algorithm.

### Use Case: Nearsightedness of lattice energies in ice structures

There are two interesting regimes for feature or sample selection – the practical reduction of data spaces for efficiently computing supervised models and the interpretability of a small handful of selected features or samples. As the former is thoroughly covered in Cersonsky *et al.* ^7^, we will focus on the latter here.

**Figure 7.**
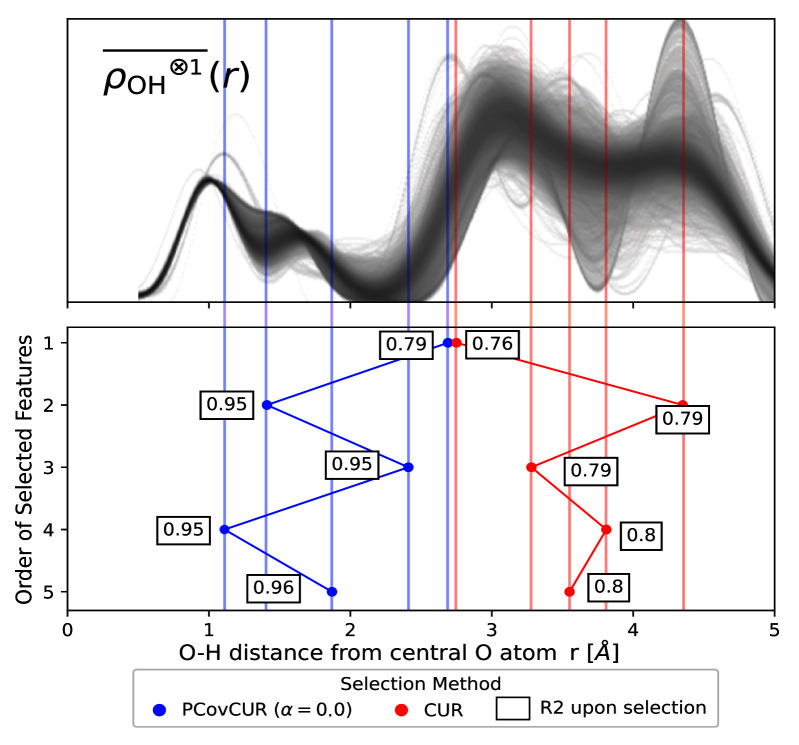
Selecting Features for Learning Lattice Energies. (Top) The real space “Radial Spectrum” $\bar{\rho_{OH}^{⊗1}}(r)$ for the hydrogen neighbours of central oxygen environments in the ice dataset. Each line corresponds to one oxygen environment. (Bottom) Selected features for both methods, noting the distance on the x-axis and selection method by colour (blue for PCovCUR and red for CUR). The number next to each point corresponds to the *R* ^2^ for the prediction of the lattice energies with a linear model upon selection of the feature.

Here we employ the real space “Radial Spectrum” expanded on the O-H distances of the central oxygen environments for the stable ice crystals. The distribution of the $\bar{\rho_{OH}^{⊗1}}(r)$ features is shown in the top panel of Figure 7.

We compare CUR with its hybrid variant PCovCUR to select 5 features. While the two selection methods choose a similar initial feature (corresponding to the hydrogen neighbours at ≈ 3.0 Å), the subsequent selected features diverge between the methods thereafter.

Within five selections, PCovCUR selects features that are capable of regressing the energies with fair accuracy, with an *R* ^2^ value of 0.96. PCovCUR selects features corresponding to the density values between the first and second (2 ^nd^ and 4 ^th^ feature), and the second and third neighbour shell (1 ^st^, 3 ^rd^ and 5 ^th^ feature). This makes sense from a physical perspective – the greatest proportion of the lattice energy in a molecular solid such as ice comes from the intermolecular interactions close to the central atom. Especially when working with highly-redundant or non-efficient features, hybrid feature selection methods provide both improved performance and can reflect physical intuition.

Conversely, the unsupervised selection method CUR preferentially chooses the far-sighted features. This is explained by the fact that unsupervised methods will aim to maximise the resulting span of the feature space, and in this representation, far-sighted features show the greatest variance. ^4^ This also explains the poorer performance of regressions built on these features, which obtain a maximum *R* ^2^ value of 0.8.

### Use Case: Choosing Features for Kernel Regression in the WHO Dataset

As shown in the previous use case, it is most effective to regress the life expectancies using a non-linear model. Here we show how feature selection mechanisms incorporating linear principal covariates yields comparable results to a recursive feature selection method based upon kernel regression.

We use the previously detailed selection schemes and compare them to Recursive Feature Selection (RFS) based upon kernel ridge regression on the training set. In the latter method, we iteratively select the feature that best improves regression performance, and this can serve as a putative “best selection” in datasets with this few features. For the FPS methods, we again choose the equivalent CUR-selected first feature to initialise the algorithm.

**Figure 8.**
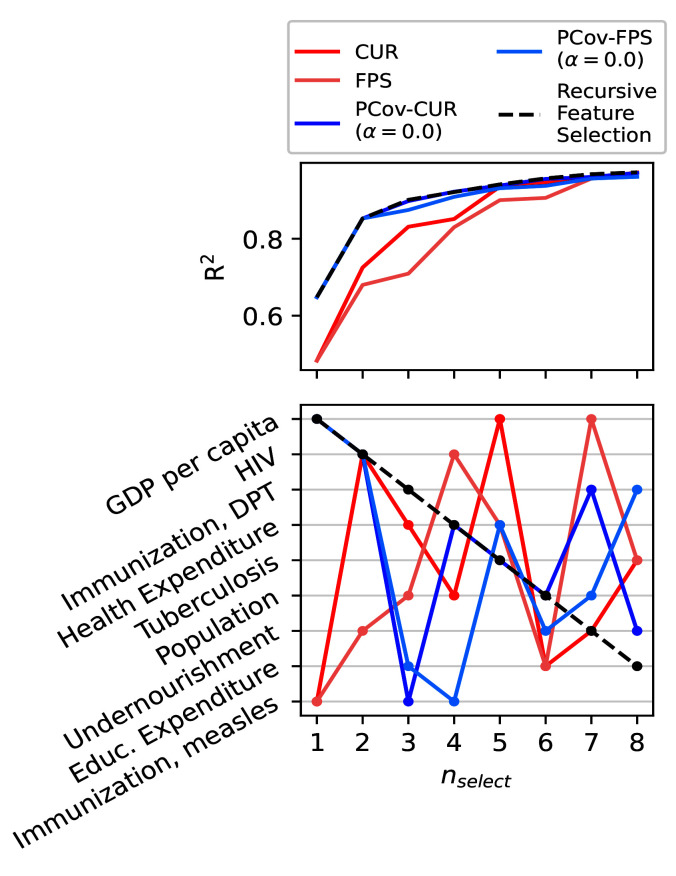
Feature Selection Methods Applied to the WHO Dataset. (top) Kernel regression performance for the different selection methods with increasing number of selected features. (bottom) Order of selected features for each selection method. We report the features in order of their selection by Recursive Feature Selection, which shows that the PCov-inspired methods most closely align with this “ground-truth”.

In each feature selection scheme, we select using the described methods and then compute the kernel ridge regression error for an offset test set. As shown in Figure 8, the parameters with greatest impact on the regression are a country’s GDP and prevalence of HIV/AIDS. This is unsurprising, as it is well-recognised in literature that the wealth of a country and prevalence of HIV/AIDS in a country directly impacts the life expectancy ^45– 47^. This is shown in the jump in accuracy for PCov-FPS and PCov-CUR at the second selection as well as for FPS and CUR at the fifth and seventh selection, respectively. Otherwise, the most successful algorithms (RFS, PCov-CUR, and PCov-FPS) also incorporate different immunisations and health expenditures.

### Directional convex hull

**Figure 9.**
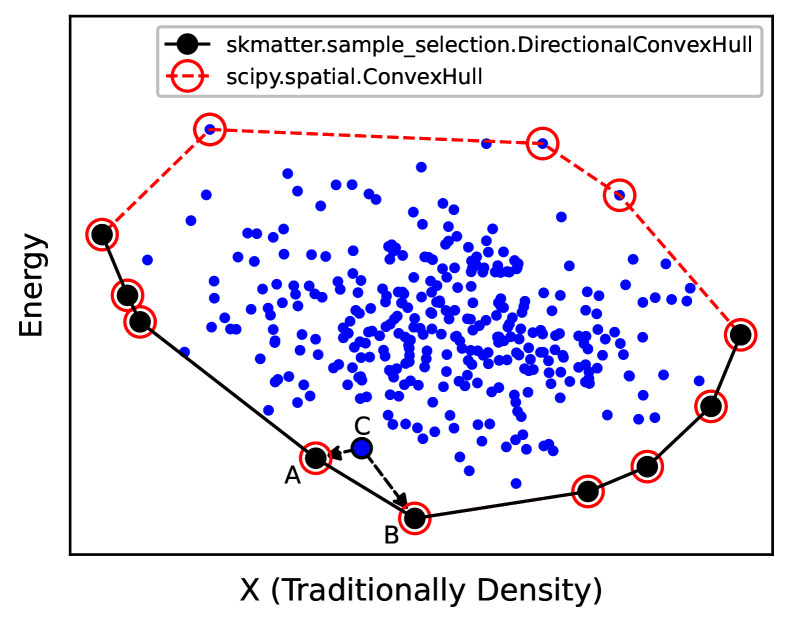
Schematic of a Convex Hull Construction. We first represent our data points across some variable (X, traditionally macroscopic density in thermodynamic convex hulls) and our energies. As shown by the black circles, our energetic convex hull lies at the bottom boundary of this plot. Programmatically, we use scipy.spatial.ConvexHull to determine the omnidirectional convex hull (red points), then select those simplices whose normal vector points negative to the energy. Any phase above the convex hull (e.g. C) will decompose into the two phases directly below it on the convex hull (e.g. A and B).

In the context of chemical sciences, a convex hull represents the set of structures that are thermodynamically stable from the perspective of a mixing model. In other words, if phases A, B, and C are of compatible stoichiometries, and A and B lie on the convex hull and phase C lies above, then C is unstable towards decomposition into a separated mixture of A and B ( Figure 9).

Usually, convex hulls are determined by a plot of the phases’ densities and energies. The convex hull is the set of points sitting on the lower boundary of this plot. Anelli *et al.* ^48^ showed that the comparative dimension can be a generalised latent variable that spans the diversity of the given dataset, in their case, a sketchmap component ^49^.

Outside of chemical sciences, a convex hull is typically considered the set of points that define a bounding manifold, i.e., the vertices of the convex shape in which all other points are contained. This is implemented in Python in the package scipy ^50^ that employs Qhull ^51^. We extend this functionality by allowing users to select the vertices subject to a directionality constraint (e.g., those points that define the surface below all other points with respect to a given observable). We have implemented this without explicit reference to chemical or energetic considerations, such that the DirectionalConvexHull can be used for similar tasks, including those shown in the mathematics ^52^ and economics ^53^ fields.

**Implementation** In scikit-matter, DirectionalConvexHull is exposed through the skmatter.sample_selection submodule. In addition to the traditional input matrix and target vector, the fit function takes as input which columns of **X** to consider in the convex hull construction, where the default behavior is to use the first column of **X**.

During the fit function, we employ scipy.spatial.ConvexHull to determine the omnidirectional convex hull. We then determine which of these points lie on simplices whose normal points downwards in the supplied property dimension. Like the selectors covered on Page 11, the indices of these points are saved into the class variable selected_idx_. Once the hull has been determined, the score_samples function determines the distance of given samples to the hull in the property dimension. One may also use the score_feature_matrix function to obtain the distance of samples to the hull in the dimensions *not* used to construct the directional convex hull. This distance in the higher-dimensional space is a measure of the putative stability of all non-hull phases, where phases near the hull are considered more stable than those further ^48^.

### Case Study: Determining the convex hull of ice structures

Engel *et al.* ^14^ determined a “generalised” convex hull that explicitly accounts for energetic and conformational uncertainty by using numerous trials of the algorithm detailed here. In each trial, they would perturb the atomic coordinates and energies of a selected number of structures within the uncertainty estimate of their calculations and compute the convex hull. They determined the convex hull as the points that are most often selected across many trials. Here, we will demonstrate one such trial by selecting the hull points using the REMatch kernel ^54^ as done in the original publication.

**Figure 10.**
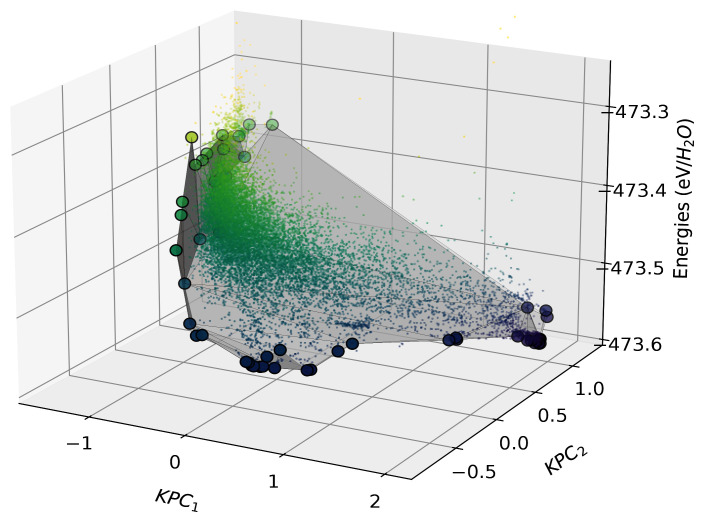
The directional convex hull of ice structures. Here we show a directional convex hull, constructed using the first two KPCA dimensions as features and the per-molecule energies of ice structures as the target property. The small points correspond to all the structures in the dataset, whereas the larger points correspond to those that lie on the convex hull (shown by the grey surface), as determined using the fit function of scikit-matter.

We build a two-component Kernel PCA on the normalised kernel matrix using the scikit-learn implementation. We construct our directional convex hull on these components, with the resulting hull shown in Figure 10.

In Figure 10, we see that the points that lie on the vertices of the directional convex hull are lower in energy relative to chemically similar points in their surroundings. As all structures that sit above the hull energetically will decompose into a mixture of the more stable phases, those on the vertices are more likely to be found in nature or be candidates for experimental synthesis. Although the KPCA features may appear abstract, they often correlate with characteristics of the structures, which are qualitatively analysable through chemiscope ^55^ or similar visualisation suites.

## Conclusions

In this work we have demonstrated the use of scikit-matter, a scikit-learn extension that focuses on functions of particular relevance in materials science and chemistry. As the examples on the WHO dataset show, the features considered need not be chemically-based correlation functions, but might be economic predictors, diagnostic test results, or the engineered features of different autoencoder infrastructures. This illustrates how the compatibility of scikit-matter with scikit-learn allows a frictionless embedding of the demonstrated implementations into data-driven workflows in any domain. This has the capability of not only introducing beneficial algorithms to users that may be unfamiliar with them but also reduces the time-consuming implementation of these methods for knowledgeable users, thereby accelerating research endeavours across a wide range of fields. The module also provides a basis for further methods developed in materials science and chemistry communities to be integrated into a stable library in the future.

## Installing scikit-matter

scikit-matter is available via the Python Package Index (PyPi) ^56^ and through GitHub. Independent developers are encouraged to contribute and can find more information on how to do so on the package documentation at scikit-matter.readthedocs.io.

## Data Availability

Materials Cloud: Mapping uncharted territory in ice from zeolite networks to ice structures. DOI: 10.24435/materialscloud:2018.0010/v1. Engel *et al.* ^16^ The project employs the following underlying data: dataset.xyz: Structures in XYZ format for 15,869 geometry-optimised ice structures. Optimisation was carried out using PBE-DFT in the CASTEP code, with a plane-wave energy cut-off of 490 eV, maximum k-point spacing of 2 *π* × 0.07 Å ^-1^, and on-the-fly generated ultrasoft pseudopotentials. properties.dat: Properties of all ice structures in dataset.xyz, including the CSD identifier, number of atoms per unit cell, density, configurational energy, and energy with respect to the energy-density convex hull. We also employ publicly available datasets provided by The World Bank: Life expectancy at birth, total (years), World Bank ^18^. Population, total, World Bank ^19^. GDP per capita (current USD), World Bank ^20^. Current health expenditure (percentage of GDP), World Bank ^21^. Government expenditure on education, total (percentage of GDP) ^22^. Prevalence of HIV, total (percentage of population ages 15–49) World Bank ^23^. Incidence of tuberculosis (per 100,000 people) World Bank ^24^. Immunization, measles (percentage of children ages 12–23 months), World Bank ^25^. Immunization, DPT (percentage of children ages 12–23 months) World Bank ^26^. Prevalence of undernourishment (percentage of population) World Bank ^27^. The aggregated WHO dataset can be retrieved through skmatter.datasets.load_who_dataset. Data are available under the terms of the Creative Commons Attribution 4.0 International license (CC-BY 4.0).

## Footnotes

^1^We optimise our basis functions starting from 100 basis functions of the DVR basis ^4^.

^2^computed using DFTB+ ^17^ with the Third-Order Parameters for Organic and Biological Systems (3OB)

^3^Greedy algorithms choose points iteratively based upon a scoring metric, whereas non-greedy algorithms simultaneously choose points that meet certain criteria.

^4^To compensate this effect, a radial scaling is often applied to SOAP features, usually leading to improved regression of atomistic properties.

## References

[1] Shapeev AV : Moment tensor potentials: A class of systematically improvable interatomic potentials. *Multiscale Model Simul.* 2016;14(3):1153–1173. 10.1137/15M1054183

[2] Drautz R : Atomic cluster expansion for accurate and transferable interatomic potentials. *Phys Rev B.* 2019;99(1):014104. 10.1103/PhysRevB.99.014104

[3] Deringer VL Bartók AP Bernstein N : Gaussian process regression for materials and molecules. *Chem Rev.* 2021;121(16):10073–10141. 10.1021/acs.chemrev.1c00022 34398616 PMC8391963

[4] Musil F Grisafi A Bartók AP : Physics-Inspired Structural Representations for Molecules and Materials. *Chem Rev.* 2021;121(16):9759–9815. 10.1021/acs.chemrev.1c00021 34310133

[5] Bartók AP De S Poelking C : Machine learning unifies the modeling of materials and molecules. *Sci Adv.* 2017;3(12):e1701816. 10.1126/sciadv.1701816 29242828 PMC5729016

[6] Willatt MJ Musil F Ceriotti M : Feature optimization for atomistic machine learning yields a data-driven construction of the periodic table of the elements. *Phys Chem Chem Phys.* 2018;20(47):29661–29668. 10.1039/c8cp05921g 30465679

[7] Cersonsky RK Helfrecht BA Engel EA : Improving sample and feature selection with principal covariates regression. *Mach Learn: Sci Technol.* 2021;2(3):035038. 10.1088/2632-2153/abfe7c

[8] Parsaeifard B De DS Christensen AS : An assessment of the structural resolution of various fingerprints commonly used in machine learning. *Mach Learn: Sci Technol.* 2021;2(1):015018. 10.1088/2632-2153/abb212

[9] Goscinski A Fraux G Imbalzano G : The role of feature space in atomistic learning. *Mach Learn: Sci Technol.* 2021;2(2):025028. 10.1088/2632-2153/abdaf7

[10] Helfrecht BA Cersonsky RK Fraux G : Structure-property maps with kernel principal covariates regression. *Mach Learn: Sci Technol.* 2020;1(4):045021. 10.1088/2632-2153/aba9ef

[11] Behler J : RuNNer. Reference Source

[12] Bartók-Pártay A Bartók-Pártay L Bianchini F : libAtoms+QUIP. 2020. Reference Source

[13] Novikov IS Gubaev K Podryabinkin EV : The MLIP package: moment tensor potentials with MPI and active learning. *Mach Learn: Sci Technol.* 2021;2(2):025002. 10.1088/2632-2153/abc9fe

[14] Engel EA Anelli A Ceriotti M : Mapping uncharted territory in ice from zeolite networks to ice structures. *Nat Commun.* 2018;9(1):2173. 10.1038/s41467-018-04618-6 29872048 PMC5988809

[15] Talirz L Kumbhar S Passaro E : Materials cloud, a platform for open computational science. *Sci Data.* 2020;7(1):299. 10.1038/s41597-020-00637-5 32901046 PMC7479138

[16] Engel EA Anelli A Ceriotti M : Mapping uncharted territory in ice from zeolite networks to ice structures. 2018. 10.1038/s41467-018-04618-6 29872048 PMC5988809

[17] Hourahine B Aradi B Blum V : DFTB+, a software package for efficient approximate density functional theory based atomistic simulations. *J Chem Phys.* 2020;152(12):124101. 10.1063/1.5143190 32241125

[18] World Bank: Life expectancy at birth, total (years).Technical report, The World Bank, Washington, DC,2023. Reference Source

[19] World Bank: Population, total.Technical report, The World Bank, Washington, DC,2023. Reference Source

[20] World Bank: Gdp per capita (current us$).Technical report, The World Bank, Washington, DC,2023. Reference Source

[21] World Bank: Current health expenditure (% of gdp).Technical report, The World Bank, Washington, DC,2023. Reference Source

[22] World Bank: Government expenditure on education, total (% of gdp).Technical report, The World Bank, Washington, DC,2023. Reference Source

[23] World Bank: Prevalence of hiv, total (% of population 15-49).Technical report, The World Bank, Washington, DC,2023. Reference Source

[24] World Bank: Incidence of tuberculosis (per 100,000 people).Technical report, The World Bank, Washington, DC,2023. Reference Source

[25] World Bank: Immunization, measles (% of children ages 12-23 months).Technical report, The World Bank, Washington, DC,2023. Reference Source

[26] World Bank: Immunization, dpt (% of children ages 12-23 months).Technical report, The World Bank, Washington, DC,2023. Reference Source

[27] World Bank: Prevalence of undernourishment (% of population). Technical report, The World Bank, Washington, DC,2023. Reference Source

[28] Bartók AP Kondor R Csányi G : On representing chemical environments. *Phys Rev B.* 2013;87(18):184115. 10.1103/PhysRevB.87.184115

[29] Prodan E Kohn W : Nearsightedness of electronic matter. *Proc Natl Acad Sci U S A.* 2005;102(33):11635–8. 10.1073/pnas.0505436102 16087868 PMC1188007

[30] Caro MA : Optimizing many-body atomic descriptors for enhanced computational performance of machine learning based interatomic potentials. *Phys Rev B.* 2019;100(2):024112. 10.1103/PhysRevB.100.024112

[31] Kermode JR : QUIP. 2008. Reference Source

[32] Csányi G Winfield S Kermode JR : Expressive programming for computational physics in fortran 95+. *IoP Comp Phys Newsletter.* 2007.

[33] Kermode JR : f90wrap: an automated tool for constructing deep python interfaces to modern fortran codes. *J Phys Condens Matter.* 2020;32(30):305901. 10.1088/1361-648X/ab82d2 32209737

[34] Himanen L Jäger MOJ Morooka EV : DScribe: Library of descriptors for machine learning in materials science. *Comput Phys Commun.* 2020;247:106949. 10.1016/j.cpc.2019.106949

[35] Ceriotti M Emsley M Paruzzo F : Chemical shifts in molecular solids by machine learning datasets. *Materials Cloud Archive.* 2019. 10.24435/materialscloud:2019.0023/v2 PMC6206069 30374021

[36] Goscinski A Fraux G Imbalzano G : The role of feature space in atomistic learning. *Mach Learn Sci Technol.* 2021;2(2):025028. 10.1088/2632-2153/abdaf7

[37] Goscinski A Musil F Pozdnyakov S : Optimal radial basis for density-based atomic representations. *J Chem Phys.* 2021;155(10):104106. 10.1063/5.0057229 34525832

[38] de Jong S Kiers HAL : Principal covariates regression: Part I. Theory. *Chemometr Intell Lab Syst.* 1992;14(1–3):155–164. 10.1016/0169-7439(92)80100-I

[39] Schölkopf B Smola A Müller KR : Nonlinear component analysis as a kernel eigenvalue problem. *Neural Computation.* 1998;10(5):1299–1319. 10.1162/089976698300017467

[40] Cersonsky TEK Cersonsky RK Saade GR : Placental lesions associated with stillbirth by gestational age, according to feature importance: results from the Stillbirth Collaborative Research Network. *Placenta.* 2023. 10.1016/j.placenta.2023.04.005 PMC10192128 37080046

[41] Mahoney MW Drineas P : CUR matrix decompositions for improved data analysis. *Proc Natl Acad Sci U S A.* 2009;106(3):697–702. 10.1073/pnas.0803205106 19139392 PMC2630100

[42] Imbalzano G Anelli A Giofré D : automatic selection of atomic fingerprints and reference configurations for machine-learning potentials. *J Chem Phys.* 2018;148(24):241730. 10.1063/1.5024611 29960368

[43] Du Q Faber V Gunzburger M : Centroidal voronoi tessellations: Applications and algorithms. *SIAM review.* 1999;41(4):637–676. 10.1137/S0036144599352836

[44] da Costa-Luis C Larroque SK Altendorf K : tqdm: A fast, Extensible Progress Bar for Python and CLI. *Zenodo.* 2022. 10.5281/zenodo.7046742

[45] Mathers CD Sadana R Salomon JA : Healthy life expectancy in 191 countries, 1999. *Lancet.* 2001;357(9269):1685–1691. 10.1016/S0140-6736(00)04824-8 11425392

[46] Ashford LS : How HIV and AIDS affect populations. *World.* 2006;1:38–600. Reference Source

[47] Hansen CW : The relation between wealth and health: Evidence from a world panel of countries. *Econ Lett.* 2012;115(2):175–176. 10.1016/j.econlet.2011.12.031

[48] Anelli A Engel EA Pickard J : Generalized convex hull construction for materials discovery. *Phys Rev Materials.* 2018;2(10):103804. 10.1103/PhysRevMaterials.2.103804

[49] Ceriotti M Tribello GA Parrinello M : Simplifying the representation of complex free-energy land-scapes using sketch-map. *Proceedings of the National Academy of Sciences.* 2011;108(32):13023–13028. 10.1073/pnas.1108486108 PMC3156203 21730167

[50] Virtanen P Gommers R Oliphant TE : SciPy 1.0: Fundamental Algorithms for Scientific Computing in Python. *Nat Methods.* 2020;17(3):261–272. 10.1038/s41592-019-0686-2 32015543 PMC7056644

[51] Barber CB Dobkin DP Huhdanpaa H : The quickhull algorithm for convex hulls. *ACM Transactions on Mathematical Software (TOMS).* 1996;22(4):469–483. 10.1145/235815.235821

[52] Liu W Yuen SY Chung KW : A general-purpose multi-dimensional convex landscape generator. *Mathematics.* 2022;10(21):3974. 10.3390/math10213974

[53] Anderson G Crawford I Leicester A : Efficiency analysis and the lower convex hull approach. Quantitative Approaches to Multidimensional Poverty Measurement. 2008;176–191. 10.1057/9780230582354_10

[54] De S Bartók AP Csányi G : Comparing molecules and solids across structural and alchemical space. *Phys Chem Chem Phys.* 2016;18(20):13754–13769. 10.1039/c6cp00415f 27101873

[55] Fraux G Cersonsky RK Ceriotti M : Chemiscope: interactive structure-property explorer for materials and molecules. *J Open Source Softw.* 2020;5(51):2117. 10.21105/joss.02117

[56] Python package index - pypi. 2003. Reference Source

