# Supplementary material for: scikit-matter : A Suite of Generalisable Machine Learning Methods Born out of Chemistry and Materials Science
Source: Open Res Eur. 2025 Dec 26;3:81. Originally published 2023 May 16. [Version 3] doi: 10.12688/openreseurope.15789.3 (PMC10792272; doi:10.12688/openreseurope.15789.3)
Supplement: Peer review history

# Reviewer report: Reviewer response for version 3

**DOI:** 10.21956/openreseurope.23357.r66960

**Published:** 2026-01-10

**Authors:** Ekaterina I. Izgorodina

**Affiliations:** 1 Monash Computational Chemistry Group, School of Chemistry, Monash University, Clayton, Victoria, Australia

The authors fully addressed the raised comments.

Is the rationale for developing the new software tool clearly explained?

Yes

Is the description of the software tool technically sound?

Yes

Are sufficient details of the code, methods and analysis (if applicable) provided to allow replication of the software development and its use by others?

Yes

Is sufficient information provided to allow interpretation of the expected output datasets and any results generated using the tool?

Yes

Reviewer Expertise:

Theoretical and computational chemistry, development of ML/AI algorithms to study thermodynamics and kinetics of chemical reactions and predict molecular and thermodynamic properties of individual molecules, large-scale systems and chemical reactions

I confirm that I have read this submission and believe that I have an appropriate level of expertise to confirm that it is of an acceptable scientific standard.

# Reviewer report: Reviewer response for version 2

**DOI:** 10.21956/openreseurope.17559.r36893

**Published:** 2024-02-21

**Authors:** Robert Meissner

**Affiliations:** 1 Hamburg University of Technology, Hamburg, Germany

Along with their manuscript, the authors provide a solid and robust software extension, fully in the spirit of the well-known scikit-learn package, and dedicated to extending those functions "that are actively used in the field of computational science and modeling of materials and chemical systems". The work presented here not only focuses on methods that are particularly relevant to materials science and chemistry, but also extends its methods to relevant data outside of the field of materials science and chemistry. Installing this extension using PIP or Anaconda is easy even for novice Pythonistas. Apart from that, a cornerstone of such an implementation is well-done documentation and relevant but easy-to-understand examples. While the indeed excellent documentation is available on the package website, the latter mentioned examples are not only illustrated very well in the manuscript, but are also provided as easy-to-use Jupyter notebooks that can be downloaded from the website.

 I have read the previous reports carefully and both have done a very thorough investigation and I do not have much to add to their review. I agree that especially the documentation is important and the authors have done a good job here, while also trying their best to make their code accessible by providing easy-to-use but relevant examples. Their brief explanation of the available methods in the manuscript is sufficient and summarizes the key principles of the underlying methods very well, while always providing additional literature for the more curious reader.

 I followed the previous discussion about more general frameworks like SMILES strings and whether they could be discussed in addition to the density-based approach present in the manuscript. SMILES and related techniques, such as SMARTS and SMIRKS, for finding patterns in molecular graphs or describing reactions, are a very active and important field in computational chemistry. However, SMILES have the disadvantage that they are not necessarily unique. Several SMILES strings represent the same molecule. This is solved by using canonical SMILES. However, they still depend on the specific canonicalization algorithm used to generate them. This can cause problems when used with natural language processing (NLP) algorithms if this redundancy is not properly handled in the raw data or if different (SMILES) data sources are used. In my opinion, the concept of SMILES should not be included in this work, as useful as they are, as they are conceptually too different from what is presented here and usually require other approaches, e.g. NLP.

 However, to perhaps answer Prof. Izgorodina's question: It is possible to use SMILES strings in a high-throughput manner, called STaGE (Small molecule Topology GEnerator), to generate reliable 3D molecular structures and calculate from those corresponding physical properties such as solvation free energies ref [1]. Perhaps this is a bit of a niche application, but as we have shown, SMILES can thus be used in combination with STaGE, SOAP and Kernel Ridge Regression methods ref [2] to predict corrosion properties based on the resulting molecular similarities. Thus, I think it is a bit unfair to conclude from the statement that SOAP is not well-known outside the field of atomistic machine learning field that this is "a very small community" and that "this feature may not be of interest to a much broader community working on the discovery of new materials".

 Leaving the important area of string representations for now, scikit-matter has the potential to make density-based approaches, e.g. SOAP, and related ML approaches more accessible to a wider community. Moreover, the techniques presented here are made available to other fields as well, since they do not necessarily have to be used in the context of density-based representations, as impressively demonstrated with the WHO data on life expectancy. This efforts of the authors to make their computational approaches more accessible to the wider community are much appreciated.

 I have a couple of minor things which might be considered:

- I wonder if the labeling of the y-axis in 2b) is correct. Is the notation GFRE 100->100 correct or is it related to the statement "The numerical accuracy of the solver is computed with GFRE for the identity relationship for 100 basis functions"? This may need further explanation.
- What is the underlying toy model of Figure 3? Perhaps it would be helpful to mention roughly what the colors in Figure 3 mean in the caption to help understand what the figure is supposed to demonstrate.
- PCovCUR, PCov-CUR, PCovFPS and PCov-FPS are not consistently used.

Is the rationale for developing the new software tool clearly explained?

Yes

Is the description of the software tool technically sound?

Yes

Are sufficient details of the code, methods and analysis (if applicable) provided to allow replication of the software development and its use by others?

Yes

Is sufficient information provided to allow interpretation of the expected output datasets and any results generated using the tool?

Yes

Reviewer Expertise:

Molecular Dynamics, Atomistic Machine Learning, Electrochemistry

I confirm that I have read this submission and believe that I have an appropriate level of expertise to confirm that it is of an acceptable scientific standard.

## References

[1] : Automatic GROMACS topology generation and comparisons of force fields for solvation free energy calculations. *J Phys Chem B* .2015;119(3) : 10.1021/jp505332p 810-23 10.1021/jp505332p 25343332

[2] : Exploring structure-property relationships in magnesium dissolution modulators. *npj Materials Degradation* .2021;5(1) : 10.1038/s41529-020-00148-z 10.1038/s41529-020-00148-z

# Reviewer report: Reviewer response for version 2

**DOI:** 10.21956/openreseurope.17559.r36900

**Published:** 2024-01-16

**Authors:** Brian DeCost

**Affiliations:** 1 National Institute of Standards and Technology, Gaithersburg, Maryland, USA

This manuscript presents an accessible and composable materials modeling interface that is compatible with a large sector of the general python-based data driven modeling ecosystem. The library currently focuses on materials-informatics-specific workflows for feature and sample selection and model evaluation, as well as high-level compatibility with the diverse set of quickly-evolving materials modeling methodologies. The manuscript is clearly presented, and the online documentation is complete, clear, and presented. The library will play an important role in helping the materials and chemical informatics community develop and apply data-driven research methods efficiently and correctly, which will have a large impact across this growing field.

Is the rationale for developing the new software tool clearly explained?

Yes

Is the description of the software tool technically sound?

Yes

Are sufficient details of the code, methods and analysis (if applicable) provided to allow replication of the software development and its use by others?

Yes

Is sufficient information provided to allow interpretation of the expected output datasets and any results generated using the tool?

Yes

Reviewer Expertise:

Computational materials science; applied machine learning

I confirm that I have read this submission and believe that I have an appropriate level of expertise to confirm that it is of an acceptable scientific standard.

# Reviewer report: Reviewer response for version 2

**DOI:** 10.21956/openreseurope.17559.r35168

**Published:** 2023-10-16

**Authors:** Andrew S. Rosen

**Affiliations:** 1 Department of Materials Science and Engineering, University of California, Berkeley, Berkeley, CA, USA

I thank the authors for their detailed and valuable response. They have addressed all of my requested (minor) changes, and I am very happy to formally "approve" this article. Thank you for the excellent contribution, which I am confident will greatly benefit the ML community!

Is the rationale for developing the new software tool clearly explained?

Yes

Is the description of the software tool technically sound?

Yes

Are sufficient details of the code, methods and analysis (if applicable) provided to allow replication of the software development and its use by others?

Yes

Is sufficient information provided to allow interpretation of the expected output datasets and any results generated using the tool?

Yes

Reviewer Expertise:

Computational materials chemistry, quantum-chemical methods, machine learning for materials discovery.

I confirm that I have read this submission and believe that I have an appropriate level of expertise to confirm that it is of an acceptable scientific standard.

# Reviewer report: Reviewer response for version 1

**DOI:** 10.21956/openreseurope.17055.r31732

**Published:** 2023-07-17

**Authors:** Ekaterina I. Izgorodina

**Affiliations:** 1 Monash Computational Chemistry Group, School of Chemistry, Monash University, Clayton, Victoria, Australia

The manuscript by Alexander Goscinski et al. “scikit-matter: A Suite of Generalisable Machine Learning Methods Born out of Chemistry and Materials Science” describes the implementation of four new features within scikit-matter that are applicable to study materials science. These features include feature reconstruction measures, Linear and Kernelized Principal Covariates Regression, Farthest Point Sampling, CUR, and their PCovR-Adaptations and Directional Convex Hull Construction. I enjoyed reading the manuscript and I think it has successfully showcased how new features can further improve the discovery of new materials. There are still several minor issue that must be addressed prior to indexing.

 All subheadings are formatted equally which is rather confusing. For each which any subsection included within the feature section could be represented differently. For example, a smaller sized and italicised font could be used instead.

 In terms of the first feature discussed in the manuscript – feature reconstruction measures – gives an example of how the number of basis functions used to represent neighbouring shells of water molecules could be much easier minimised by using this feature. The feature is based around the SOAP representation of a chemical system, in which each atom in the system is represented with a Gaussian density. I was wondering if a more general example could be given here. As the authors pointed out themselves, the SOAP representation is not well known outside of a very small community and therefore, this feature may not be of interest to a much broader community working on the discovery of new materials. Majority of scientists in the field use the smiles string representation to predict descriptors to be used in the discovery process. I was wondering if this feature could be used in combination with the smiles string to generate a density-based representation of the system.

 Figure 5 is a little confusing. The authors argue that the combination of the PCovR kernel can distinguish among varying oxygen environments – hydroxide, hydronium cation and different arrangements of water. Can the three environments be elaborated and accompanied with corresponding figures that showcase difference in the oxygen environments. Equally important, it should be clearly stated how these environments were selected and why Mulliken charges were used for this purpose. I am a little hesitant about a wider applicability of Mulliken charges as this is one of the most approximate schemes to predict partial atomic charges and strongly relies on the basis set used. The last point should be comment on in the manuscript.

 Figure 8 is difficult to understand. It took me a while to realise that essentially the order of the parameters on the y-axis are given with respect to the best performing model proposed in the manuscript – Recursive Feature Selection. The “jagged” trends for other models are slightly confusing as the clearly favoured a different order of the parameters. I was wondering gif there was a better and clear way to represent these data in a Figure.

Is the rationale for developing the new software tool clearly explained?

Yes

Is the description of the software tool technically sound?

Yes

Are sufficient details of the code, methods and analysis (if applicable) provided to allow replication of the software development and its use by others?

Yes

Is sufficient information provided to allow interpretation of the expected output datasets and any results generated using the tool?

Yes

Reviewer Expertise:

Theoretical Chemistry, Molecular Dynamics and Machine Learning

I confirm that I have read this submission and believe that I have an appropriate level of expertise to confirm that it is of an acceptable scientific standard, however I have significant reservations, as outlined above.

# Response

**Published:** 2023-08-25

**Authors:** Alexander Goscinski

**Affiliations:** Institute of Materials, Ecole Polytechnique Federale de Lausanne, Lausanne, Vaud, Switzerland

**The manuscript by Alexander Goscinski et al. “scikit-matter: A Suite of Generalisable Machine Learning Methods Born out of Chemistry and Materials Science” describes the implementation of four new features within scikit-matter that are applicable to study materials science. These features include feature reconstruction measures, Linear and Kernelized Principal Covariates Regression, Farthest Point Sampling, CUR, and their PCovR-Adaptations and Directional Convex Hull Construction. I enjoyed reading the manuscript and I think it has successfully showcased how new features can further improve the discovery of new materials. There are still several minor issue that must be addressed prior to indexing.**

 We thank Prof. Izgorodina for her kind words, and are happy that she finds the publication useful!

**All subheadings are formatted equally which is rather confusing. For each which any subsection included within the feature section could be represented differently. For example, a smaller sized and italicised font could be used instead.**

 We changed the structure of the paper by adding a numeration to the sections as well as removing the method section to move all features one level higher. For example, the methods “FPS” and “CUR” are now subsubsections so that the paragraph “Workflow” can be clearly identified as part of the corresponding method.

**In terms of the first feature discussed in the manuscript – feature reconstruction measures – gives an example of how the number of basis functions used to represent neighbouring shells of water molecules could be much easier minimised by using this feature. The feature is based around the SOAP representation of a chemical system, in which each atom in the system is represented with a Gaussian density. I was wondering if a more general example could be given here. As the authors pointed out themselves, the SOAP representation is not well known outside of a very small community and therefore, this feature may not be of interest to a much broader community working on the discovery of new materials. Majority of scientists in the field use the smiles string representation to predict descriptors to be used in the discovery process. I was wondering if this feature could be used in combination with the smiles string to generate a density-based representation of the system.**

 The main benefit of SMILES strings is to leverage advances in natural-language processing for chemical systems. While there are possibilities for scikit-matter to support NLP-like functionalities, currently scikit-learn is not the primary package used for NLP workflows, and therefore we wouldn’t necessarily argue that scikit-matter should first-and-foremost cater to NLP models. That being said, there are many ways to embed SMILES strings into numeric representations, and, in this context, we agree with the reviewer that we could make an effort to include the greater community in these case studies. We have added some notes to sections on how SMILES-like workflows could be augmented with these methods: *Throughout the paper we use the Smooth Overlap of Atomic Positions (SOAP) representation to numerically encode our chemical data\cite{soap}. We note however, that other numerical encodings of chemical information are compatible with the methods presented here\cite{capecchi_one_2020}.* Unfortunately, SMILES strings often lack the configurational information to generate a density-based representation, and there is an active, non-trivial effort in the field to predict thermodynamically-sensible configurations from connectivity information (e.g. SMILES), see DOIs 10.1039/d0cp04670a and 10.48550/arXiv.2106.07802. We feel that in order to include a useful example based on SMILES-type descriptors we would have to focus on the methodology to translates them into continuous features, which is, in our view, a pre-processing step that is orthogonal to the methods that are currently included in the package.  

**Figure 5 is a little confusing. The authors argue that the combination of the PCovR kernel can distinguish among varying oxygen environments – hydroxide, hydronium cation and different arrangements of water. Can the three environments be elaborated and accompanied with corresponding figures that showcase difference in the oxygen environments**

 We thank Prof. Izgorodina for her comment – Figure 5 is meant to showcase the difference in chemical analyses between PCA and PCovR; PCovR can incorporate target information into structure-property maps, whereas PCA will be entirely chemically-determined. The importance here is not the environments themselves, but what we would consider “similar” environments, as visualization and mapping techniques often aim to represent similar data points nearby each other in latent space. When aiming to identify QSPR relationships, PCA is often invoked; however, PCovR is directly suited to this task, as sometimes, mappings which best represent chemical similarity (such as here) do not reflect similarity in properties as well. We invite the reviewer to explore herself the difference in the relationship using the interactive demo https://chemiscope.org/?load=https://github.com/lab-cosmo/skmatter-ore/raw/main/paper/chemiscope-oxygen-ice-structures-small.json.gz

**Equally important, it should be clearly stated how these environments :were selected and why Mulliken charges were used for this purpose. I am a little hesitant about a wider applicability of Mulliken charges as this is one of the most approximate schemes to predict partial atomic charges and strongly relies on the basis set used. The last point should be comment on in the manuscript.**

 Here we use Mulliken charges not to make any larger scientific claim, but rather to provide a reasonable and easily-understandable target quantity for PCovR for a general audience. Please see our comments to Prof. Rosen on the exact computation and provenance of these quantities.  

**Figure 8 is difficult to understand. It took me a while to realise that essentially the order of the parameters on the y-axis are given with respect to the best performing model proposed in the manuscript – Recursive Feature Selection. The “jagged” trends for other models are slightly confusing as the clearly favoured a different order of the parameters. I was wondering gif there was a better and clear way to represent these data in a Figure.**

 During the creation of the Figure we tried different orders of the y-axis and came to the conclusion that the order corresponding to the best regression performance is the clearest as this gives an interpretable importance score of the features. As the red methods are purely based on unsupervised information, it is clear that the order does not need to be correlated with RFE, therefore resulting in the “jagged” trend. We explained this in the text more precisely *The relationship between the selectors becomes more apparent the similarity is plotted using the weighted Kendall's $\tau$\cite{shieh_weighted_1998} as illustrated in \figref{fig:who_sel}b). A strong similarity is observed between the selector and their PCovR adaption (e.g. CUR and PCovCUR) as well as among the supervised selectors (PCovCUR, PCovFPS and RFA). While the supervised selectors incorporate the same information of the targets to assess feature importance, the unsupervised selectors rely on different mathematical assumptions for assessment, resulting in lower similarity.* On the other hand the feature selection schemes that are based solely on the target information (blue) are quite strongly correlating with the recursive feature selection. We updated the figure so this can be seen much easier. For that we changed the model to a linear model to remove the effect of nonlinearities in the feature selection process. In addition we added a heatmap of similarity between the selected features of the selector using the selected order of the corresponding feature selection method as rank. In this heatmap the correlation can be seen clearly.

# Reviewer report: Reviewer response for version 1

**DOI:** 10.21956/openreseurope.17055.r31730

**Published:** 2023-06-19

**Authors:** Andrew S. Rosen

**Affiliations:** 1 Department of Materials Science and Engineering, University of California, Berkeley, Berkeley, CA, USA

# Overall Comments

 In their submitted work, Goscinski et al. have described and released `scikit-matter`, a Python package built around the `scikit-learn` API that provides a suite of feature selection/analysis tools that are likely to be of particular interest to the computational materials science community. After going through the paper and corresponding code, I am confident that this work warrants acceptance in Open Research Europe. To the best of my knowledge, there is no comparable package published to date, and the code itself fills a niche between pure ML regression codes like `scikit-learn` and ML featurization codes like `QUIP` and `DScribe`.

 In short, I recommend this work for acceptance in Open Research Europe with only minor revisions, mainly focused on making the documentation more accessible to a reader who may not (yet) be familiar with the underlying techniques and perhaps has not (yet) read the corresponding paper. I also have a few very minor comments and questions about the paper, as described below.

 Congratulations to the authors on this nice work, and I look forward to seeing `scikit-matter` continue to evolve in the future.

 # The Paper

 ## Overall Comments

 Overall, I found the paper to be excellent. I learned a lot from it, and I thought it was exceptionally clear. I found the examples to be very insightful, such as the convergence test for the number of basis functions and the convex hull analysis broken down by the principal components. The paper (and corresponding code) strike a unique and admittedly challenging balance between being domain-agnostic yet also catering to methods that are likely to be especially useful to computational material scientists. I do not have any major comments on the paper, which I think will be a very useful reference for machine learning practitioners and students alike.

 ## Feature Reconstruction Measures

 This section was very clear and effective! As someone who has not used feature reconstruction measures before, one of the questions I had when reading the text was the following: "for a given dataset of interest, wouldn't doing a standard evaluation of the model cross-validation error for two separate representations also tell me which is more information-rich?" With this in mind, perhaps it might be worth taking a sentence to explain the major benefit of the feature reconstruction method approach. Is it simply that it's a more direct measure of the ability of the representation to reproduce the true data?

 The authors state that "As most interesting applications use large feature vectors, we implemented a custom RidgeRegression2FoldCV in the skmatter.linear_model module to improve computational efficiency." This immediately made me think about SOAP and how, particularly over a dataset spanning much of the periodic table in terms of unique elements, the feature vectors can be absolutely enormous. This often makes something like kernel ridge regression (and, therefore, the hyperparameter tuning process) very memory- and compute-intensive. Could a method like GFRE be used to narrow down the range of SOAP-based hyperparameters to search over by plotting a convergence curve like in Figure 2? Is that the primary use case?

 ## Principal Covariates Regression

 I thought this section was great, and I don't have any major comments. The demonstration of how PCovR can be very beneficial in KRR (particularly with non-linear kernel functions) was very insightful.

 ## FPS, CUR, and Hulls

 Again, I thought this final section was written very clearly. I have one extremely minor comment. On the bottom-left of Page 13, the text states "As all structures that sit above the hull energetically will decompose into a mixture of the more stable phases...". Technically, the convex hull is based solely on thermodynamics and therefore can only describe thermodynamic (in)stability, if I understand right. Therefore, if the kinetic barrier is high for the transition, it's not necessarily guaranteed that the structure will decompose (i.e. the structure could be meta-stable).

 ## Methods

 - Could you please include the version of the DFTB+ code that was used to generate the Mulliken partial charges? Are there any other details that need to be included here for reproducibility purposes, such as chosen Slater-Koster files?

 - What code was used to generate the features in the paper? In particular, I know that different codes have implemented SOAP differently, so for the sake of reproducibility, it would be beneficial to know any pertinent details (e.g. for reproducing Figure 2).

 ## Citations

 The citations are thorough and appropriate. You may also wish to consider the following:

 - You may wish to cite `scikit-learn` since `scikit-matter` is so heavily built around it. See https://scikit-learn.org/stable/about.html#citing-scikit-learn for the citation information.

 - You may wish to cite Bartel, _J. Mater. Sci._ (2022) https://link.springer.com/article/10.1007/s10853-022-06915-4 in the convex hull section since most people aren't very familiar with convex hull analyses and the Bartel paper is extremely accessible to a broad audience in case the reader wishes to learn more.

 # The Code

 ## GitHub Repo

 Everything looks great here! I'm very happy to see the significant test coverage. Kudos! The Python package has all the ingredients for a robust and well-maintained code.

 ## Installation

 Everything worked as expected!

 That said, I do have one very minor suggestion. Although it's in the `pyproject.toml` file, less experienced Pythonistas may not be aware of what versions of Python are supported by `scikit-matter`. So, it might be worth either mentioning that in the docs or adding the associated [PyPI badge]( https://shields.io/) to the `README.md`.

 ## Documentation

 Overall, I am very pleased by the documentation! My main suggestion has to do with the fact that most users will likely check out the code/docs before ever opening the paper, and right now the documentation isn't sufficient to explain to the user why this code might be for them. For instance, perhaps someone stumbled upon the code on GitHub after someone else star'd it. Without reading the paper, they are going to see "PCov-FPS extends upon FPS much like PCov-CUR does to CUR" on the "What's in sckit-matter" page and likely be confused as to what this means and may doubt that this is a relevant package for them.

 Thankfully, this is an easy fix in my opinion. The paper itself is extremely clear and has the key components for what I would also like to see in the docs: a brief overview of each technique and why it might be useful, along with a minimal working example for each. You can simply adapt snippets of text and/or figures from your paper straight into the docs and it'd likely be perfect. This would best be done in a new "Methods" or "Theory" section, perhaps mimicking the section for "Descriptors" in the [DScribe]( https://singroup.github.io/dscribe/latest/tutorials/tutorials.html#descriptors) package.

 There are admittedly some details in the API Reference section already, but it's somewhat technical. The API Reference section is also really meant for current users of the code who want to look up the details of a particular function/class. It's not really meant for new users who are trying to figure out the methodology, which is why I think the Python package would be best served by having an additional section in the documentation dedicated to this.

 Overall, I would say this is probably the most important comment I have about the submitted work that I hope to see addressed in a future (minor) revision.

 ### Examples

 The examples in the documentation are great, and the ability to directly download the code as a Jupyter Notebook is very welcome. As a suggestion for further improvement, I think it would be especially helpful to provide a few sentences of context at the start of each example to orient the user about what the goal of the case-study is. Setting the stage a bit and defining the scope would go a long way, as it wasn't immediately clear to me what the goal was in each example until I completed it. Like above, I also think users who haven't read the paper or references therein likely won't know what PCovR means, for instance, and therefore won't immediately know which examples are relevant to their interests/needs. Providing some background to motivate things at a higher level would help close this gap.

 ### Contributing/References

 Everything is very clear here in terms of guidelines! Same for the references. I have no suggestions here.

 ### Miscellaneous

 In case the authors are curious, I made a fork of the repo and ran the codebase through several GitHub-based code analyzers, including [Deepsource]( https://deepsource.io), [Codacy]( https://app.codacy.com/), and [Sourcery](https://sourcery.ai/), to see if there were any glaring bugs or major issues. Good news --- I didn't see anything crucial! Most of the hits were stylistic in nature. This isn't too surprising given the high test coverage. Nice!

Is the rationale for developing the new software tool clearly explained?

Yes

Is the description of the software tool technically sound?

Yes

Are sufficient details of the code, methods and analysis (if applicable) provided to allow replication of the software development and its use by others?

Yes

Is sufficient information provided to allow interpretation of the expected output datasets and any results generated using the tool?

Yes

Reviewer Expertise:

Computational materials chemistry, quantum-chemical methods, machine learning for materials discovery.

I confirm that I have read this submission and believe that I have an appropriate level of expertise to confirm that it is of an acceptable scientific standard.

## References

[1] : Review of computational approaches to predict the thermodynamic stability of inorganic solids. *Journal of Materials Science* .2022;57(23) : 10.1007/s10853-022-06915-4 10475-10498 10.1007/s10853-022-06915-4

# Response

**Published:** 2023-08-25

**Authors:** Alexander Goscinski

**Affiliations:** Institute of Materials, Ecole Polytechnique Federale de Lausanne, Lausanne, Vaud, Switzerland

**Answer to Andrew S. Rosen**

**# Overall Comments**

**In their submitted work, Goscinski et al. have described and released `scikit-matter`, a Python package built around the `scikit-learn` API that provides a suite of feature selection/analysis tools that are likely to be of particular interest to the computational materials science community. After going through the paper and corresponding code, I am confident that this work warrants acceptance in Open Research Europe. To the best of my knowledge, there is no comparable package published to date, and the code itself fills a niche between pure ML regression codes like `scikit-learn` and ML featurization codes like `QUIP` and `DScribe`.**

**In short, I recommend this work for acceptance in Open Research Europe with only minor revisions, mainly focused on making the documentation more accessible to a reader who may not (yet) be familiar with the underlying techniques and perhaps has not (yet) read the corresponding paper. I also have a few very minor comments and questions about the paper, as described below.**

**Congratulations to the authors on this nice work, and I look forward to seeing `scikit-matter` continue to evolve in the future.**

 We thank Prof. Rosen for his kind words, and are happy that he finds the publication suitable for ORE.  

**# The Paper**

**## Overall Comments**

**Overall, I found the paper to be excellent. I learned a lot from it, and I thought it was exceptionally clear. I found the examples to be very insightful, such as the convergence test for the number of basis functions and the convex hull analysis broken down by the principal components. The paper (and corresponding code) strike a unique and admittedly challenging balance between being domain-agnostic yet also catering to methods that are likely to be especially useful to computational material scientists. I do not have any major comments on the paper, which I think will be a very useful reference for machine learning practitioners and students alike.**

 Again, we thank Prof. Rosen for his kind words.  

**## Feature Reconstruction Measures This section was very clear and effective! As someone who has not used feature reconstruction measures before, one of the questions I had when reading the text was the following: "for a given dataset of interest, wouldn't doing a standard evaluation of the model cross-validation error for two separate representations also tell me which is more information-rich?" With this in mind, perhaps it might be worth taking a sentence to explain the major benefit of the feature reconstruction method approach. Is it simply that it's a more direct measure of the ability of the representation to reproduce the true data?**

 It is correct that applying the feature reconstruction error (FRE) on a target property as ground truth is equivalent to a regression. One extension of the FRE is to use an arbitrary set of features as ground truth as it is more suitable to compare features when doing a quantitative assessment of different featurizations of the same representations (e.g. different radial basis functions). We have added the following to the manuscript to clarify this point: The feature set that results in the higher reconstructive measure is considered higher in information capacity (e.g. if FRM(ℱ, G) > FRM(G, ℱ), then ℱ is the more information-rich feature set). The advantage of the FRM is that it can give quantitative results about the shared information content between two sets of features, even without the existence of a ground truth. The simplest FRM is the global feature reconstruction error (GFRE), expressed as the linearly decodable information, as given by performing ridge regression between the two sets of features. We note that in the case of choosing the property as ground truth, the GFRE is equivalent to a regression task on the standardized property.   

**The authors state that "As most interesting applications use large feature vectors, we implemented a custom RidgeRegression2FoldCV in the skmatter.linear_model module to improve computational efficiency." This immediately made me think about SOAP and how, particularly over a dataset spanning much of the periodic table in terms of unique elements, the feature vectors can be absolutely enormous. This often makes something like kernel ridge regression (and, therefore, the hyperparameter tuning process) very memory- and compute-intensive. Could a method like GFRE be used to narrow down the range of SOAP-based hyperparameters to search over by plotting a convergence curve like in Figure 2? Is that the primary use case?**

 Prof. Rosen is correct that this is one beneficial use case for GFRE; optimizing the regularization parameter over a large grid but no other hyperparameters. We have added the following statements to the manuscript: In \sklearn an implementation of the leave-one-out CV with the similar purpose of speeding up the CV exists. A detailed comparison to this method can be found in one of the examples which are included in the documentation of \skmat \url{https://scikit-matter.readthedocs.io/en/v0.2.0/examples/regression/Ridge2FoldCVRegularization.html}.   that includes a new sphinx gallery example in the documentation with more detailed analysis.  

**On the bottom-left of Page 13, the text states "As all structures that sit above the hull energetically will decompose into a mixture of the more stable phases...". Technically, the convex hull is based solely on thermodynamics and therefore can only describe thermodynamic (in)stability, if I understand right. Therefore, if the kinetic barrier is high for the transition, it's not necessarily guaranteed that the structure will decompose (i.e. the structure could be meta-stable).**

 It is true that kinetics can be an obstacle to spinodal decomposition – convex hulls are typically used to assess thermodynamic processes, as opposed to kinetic ones. We have added the phrase “From a thermodynamic standpoint,” at the beginning of this sentence to clarify the physics here.  

**Could you please include the version of the DFTB+ code that was used to generate the Mulliken partial charges? Are there any other details that need to be included here for reproducibility purposes, such as chosen Slater-Koster files?**

 To the best of our knowledge, these charges were computed using DFTB+ version 21.2/22.1 and ase version 3.22.1. We apologize for the uncertainty, as an untimely computer crash has left some ambiguity in the DFTB+ versioning. DFTB+ changes between these two versions would have no impact on the ultimate computations. We used the 3ob Slater-Koster files downloaded from the dftbplus repository and the following code to generate all charges for a given frame from the ice dataset:   from ase.units import Hartree, Bohr from ase.calculators.dftb import Dftb calc = Dftb(                 atoms=frame,                 label=directory + "/dftb".format(frame.info["name"]),                 Hamiltonian_MaxAngularMomentum_="",                 Hamiltonian_MaxAngularMomentum_O="p",                 Hamiltonian_MaxAngularMomentum_H="s",                 kpts=(1, 1, 1),                 Hamiltonian_SCC="Yes",                 Hamiltonian_MaxSCCIterations=1000,                 Hamiltonian_Charge=0.0,                 Hamiltonian_DampXH="Yes",                 Hamiltonian_DampXHExponent=4.0,                 Hamiltonian_HubbardDerivs_="",                 Hamiltonian_HubbardDerivs_O=-0.1575,                 Hamiltonian_HubbardDerivs_H=-0.1857,                 Hamiltonian_ThirdOrderFull="Yes", ) frame.calc = calc calc.calculate(frame) frame.arrays["charges"] = frame.get_charges()  

 This code, as well as all codes (including those written below) used to generate figures in the paper, has been archived at https://github.com/lab-cosmo/skmatter-ore. This link has been added to the “Data Availability” section Data are available under the terms of the \href{https://creativecommons.org/licenses/by/4.0/}{Creative Commons Attribution 4.0 International license} (CC-BY 4.0). The scripts together with the required software packages used to generate the figures are available in the repository https://github.com/lab-cosmo/skmatter-ore in the form of executed jupyter notebooks with output.

**What code was used to generate the features in the paper? In particular, I know that different codes have implemented SOAP differently, so for the sake of reproducibility, it would be beneficial to know any pertinent details (e.g. for reproducing Figure 2).**

 We used librascal (github.com/lab-cosmo/librascal, commit 4e8f9fb) to compute the SOAP features using the following code. We included in the manuscript a link to the repository that contains the notebooks used for the computation of all figures. The specific hypers for each figure can be found in the corresponding notebooks You can find the hypers of Figure 2 in the following notebook https://github.com/lab-cosmo/skmatter-ore/blob/main/paper/atomrings.ipynb 

**You may wish to cite `scikit-learn` since `scikit-matter` is so heavily built around it. See https://scikit-learn.org/stable/about.html#citing-scikit-learn for the citation information. Thank you for the advice, a needed citation hidden-in-plain-sight!**

**You may wish to cite Bartel, J. Mater. Sci. (2022) https://link.springer.com/article/10.1007/s10853-022-06915-4 in the convex hull section since most people aren't very familiar with convex hull analyses and the Bartel paper is extremely accessible to a broad audience in case the reader wishes to learn more.**

 Thanks for the suggestion! We have been sure to add in this appropriate citation.  

**That said, I do have one very minor suggestion. Although it's in the `pyproject.toml` file, less experienced Pythonistas may not be aware of what versions of Python are supported by `scikit-matter`. So, it might be worth either mentioning that in the docs or adding the associated [PyPI badge]( https://shields.io/) to the `README.md`.**

 We updated the metadata of our package and it is now visible on pypi. We also specify what OS and Python version we perform the CI on (the suggestion resulted in the PR https://github.com/scikit-learn-contrib/scikit-matter/pull/200)  

**The paper itself is extremely clear and has the key components for what I would also like to see in the docs: a brief overview of each technique and why it might be useful, along with a minimal working example for each. You can simply adapt snippets of text and/or figures from your paper straight into the docs and it'd likely be perfect. This would best be done in a new "Methods" or "Theory" section, perhaps mimicking the section for "Descriptors" in the [DScribe]( https://singroup.github.io/dscribe/latest/tutorials/tutorials.html#descriptors) package.**

 We thank Prof. Rosen for the nice comment and have made appropriate changes to the documentation by adding a “Getting started” section, see https://scikit-matter.readthedocs.io/en/latest/getting-started.html   

**I think it would be especially helpful to provide a few sentences of context at the start of each example to orient the user about what the goal of the case-study is. Setting the stage a bit and defining the scope would go a long way, as it wasn't immediately clear to me what the goal was in each example until I completed it. Like above, I also think users who haven't read the paper or references therein likely won't know what PCovR means, for instance, and therefore won't immediately know which examples are relevant to their interests/needs. Providing some background to motivate things at a higher level would help close this gap.**

 Thanks for the comment, we hope with the documentation overhaul we meet the criteria of Prof. Rosen https://scikit-matter.readthedocs.io/en/latest/index.html We put a lot of effort into making it more friendly to less experienced users.
